# Thermal Image-Based Artificial Neural Network Approach to Determine Mastitis Detection in Holstein Dairy Cattle

**DOI:** 10.3390/ani16071048

**Published:** 2026-03-30

**Authors:** Hasan Alp Şahin, Edit Mikó, Hasan Önder, Wissem Baccouri

**Affiliations:** 1 Hemp Research Institute, Ondokuz Mayis University, 55139 Samsun, Türkiye; alp.sahin@omu.edu.tr; 2 Institute of Animal Science and Wildlife Management, Faculty of Agriculture, University of Szeged, 6800 Hódmezővásárhely, Hungary; baccouri.wissem@szte.hu; 3 Department of Animal Science, Faculty of Agriculture, Ondokuz Mayis University, 55139 Samsun, Türkiye; honder@omu.edu.tr

**Keywords:** thermal image, mastitis detection, Holstein, dairy cattle, artificial intelligence

## Abstract

Mastitis, a disease associated with milk production with multiple etiologies, causes significant economic losses among dairy farmers worldwide. This study aimed to detect mastitis using thermal images of the udder obtained during the milking phase from 500 Holstein dairy cows with the aid of an Artificial Neural Network (ANN). Mastitis levels were classified based on the California Mastitis Test (CMT) scores using somatic cell count (SCC) as the output variable. The dataset was divided into training (70%), validation (15%), and test (15%) subsets. RGB (Red, Green, Blue) thermal images were used to construct the input matrices. The model achieved correlation coefficients (R) of 0.91, 0.97, and 0.97 for the training, validation, and test datasets, respectively. The close agreement between validation and test performances indicates the absence of overfitting and demonstrates strong generalization capability of the proposed model. These findings suggest that artificial neural networks combined with thermal imaging can provide high-quality and reliable results for mastitis detection.

## Simple Summary

Mastitis is a common disease in dairy cows that reduces milk production and causes economic losses for farmers worldwide. Early detection is important to protect animal health and improve farm efficiency. This study examined whether thermal images taken from cow udders during milking can be used to detect mastitis using artificial intelligence. Thermal images from 500 dairy cows were analysed, and the developed model successfully distinguished between healthy and mastitis-affected udders. The consistent performance of the model showed that it could reliably analyse new data without overfitting. These results indicate that thermal imaging combined with artificial intelligence is a practical, non-invasive method for mastitis detection and may support better herd management and animal welfare.

## 1. Introduction

Increasing global demand for dairy products has driven efforts to improve milk yield through genetic selection and enhanced management practices. However, these intensification strategies have also increased the susceptibility of dairy cattle to various diseases [1]. One of the most important diseases affecting dairy cattle is mastitis [2,3]. Mastitis is an inflammation of the mammary gland, most commonly caused by bacterial infection, and is characterised by swelling, heat, pain, and hardness of the udder, reduced milk production, and an increase in somatic cell count (SCC) [4]. In addition, proper milking hygiene, biosecurity measures, and comprehensive mastitis control programmes are necessary to reduce subclinical mastitis (SCM) and prevent the spread of pathogens [5]. Mastitis has significant economic importance in dairy farming, particularly in high-yielding dairy cows, due to its negative impact on milk production and animal health, as well as the need to discard milk during treatment and withdrawal periods and the costs associated with antibiotic therapy [6]. Moreover, the use of antibiotics in mastitis treatment raises concerns regarding antimicrobial resistance and potential risks to public health if residues enter the food chain [7].

A prolonged decrease in milk yield represents a significant portion of the reduction in profitability caused by bovine mastitis. This condition is caused by bacteria such as *Staphylococcus aureus*, non-aureus staphylococci (NAS), *Escherichia coli*, *Corynebacterium bovis*, *Streptococcus uberis*, and *Streptococcus dysgalactiae*. Minor pathogens can also contribute to reduced milk yield. Among these pathogens, *E. coli* is particularly important, and controlling *E. coli*-associated mastitis in dairy farms can significantly increase milk production [8,9,10].

Mastitis is divided into two categories based on its course: clinical and subclinical. In practical dairy production, subclinical mastitis (SCM) is considerably more prevalent than clinical mastitis (CM), largely due to its subtle presentation and long latency period. Estimates suggest that for every clinically apparent case of mastitis, there may be dozens of subclinical cases, representing the majority of bovine mastitis incidents. While affecting mammary tissue and milk composition, SCM is not detectable through visual or clinical examination, resulting in a significantly high prevalence within herds [8,11].

Clinical cases account for 20–30% of mastitis-related losses, whereas subclinical cases contribute 70–80%. In approximately 90–95% of cases, the milk and udder appear normal; however, SCC increases, and milk quality and yield decline. Mastitis may persist for extended periods and, if milk from affected cows is used for calf feeding, it can negatively impact calf health and development [12].

The financial impact of mastitis primarily arises from reduced milk yield (61–70%), herd replacement (11–22%), and treatment costs (3–9%). In addition, infected animals are often culled to prevent the spread of infection. These measures, along with medication, veterinary services, labour costs, and culling further contribute to overall losses [13].

SCC is influenced by multiple factors, including the cow’s age, breed, parity, and milk yield, as well as anatomical and physiological characteristics of the udder. Environmental and management conditions, such as stress, season, nutrition, housing, and milking technique, also play important roles, along with the presence of mastitis [14,15,16,17,18,19,20,21,22,23].

In healthy cows, SCC is typically below 200,000 cells/mL, but it rises immediately after pathogens invade the mammary tissue, exceeding 1,000,000 cells/mL in cases of acute mastitis [8,24].

Advancing technologies provide new approaches that enable more sensitive and reliable identification of mastitis pathogens and inflammation-related biomarkers. Moreover, as the scale of dairy farming operations increases, the demand for alternative, rapid, and reliable diagnostic methods continues to grow [25]. In this context, the detection methods of dairy cow mastitis have made rapid progress, including SCC, California mastitis test (CMT), and electrical conductivity, which can detect dairy mastitis relatively well [26]. In addition to these standard diagnosing methods, the use of thermal camera has also been explored for this purpose [27]. With the advancements in statistical and computational techniques, several artificial intelligence-based methods have been proposed for mastitis detection [26,28].

Artificial intelligence methods, widely applied in technological fields, have increasingly been used in animal husbandry in recent years, including for the detection of mastitis. For example, principal component analysis, linear discriminant analysis, time series analysis, and Kalman filter methods were used for the diagnosis of subclinical and clinical mastitis, while fuzzy logic and artificial neural networks (ANNs) have been reported as the most effective approaches [29,30,31,32,33]. Heald et al. [30] collected milk samples from individual udder lobes and used data from the Dairy Cattle Development Agency (DHIA) in their study. Their results showed that artificial neural networks (ANNs) provide satisfactory performance in datasets with a high incidence of clinical mastitis. Heald reported that the probability of correct mastitis using ANNs varies between 57% and 71%, whereas linear discriminant analysis applied to the same data was less effective, with accuracy ranging from 42% to 57%. Although mastitis is a visible disease, SCM, considered its early stage, cannot be detected visually. Even though SCC is crucial in mastitis detection, the requirement to process milk in a laboratory environment leads to both time delays and increased costs, particularly for large herds.

Hovinen et al. [27] used thermal camera images to detect clinical mastitis. They found that the thermal camera successfully identified a 1 to 1.5 °C increase in udder skin temperature associated with clinical mastitis in all cows, as the temperature of the udder skin in the experimental and control quarters rose in parallel with rectal temperature according to ANOVA results. Gayathri et al. [34] used infrared thermography and multivariate linear models to detect seasonal clinical mastitis, finding a significant difference in udder skin temperature between affected and healthy quarters. In a follow-up study, they evaluated the effectiveness of infrared thermography for detecting both subclinical (SCM) and clinical mastitis (CM) in lactating Murrah buffaloes. Pre- and post-milking thermograms of the udder, teat, and short milk tube (SMT) surface temperatures were compared with CMT and SCC results. SMT and udder skin temperatures were significantly higher in SCM and CM quarters (*p* < 0.05), showing strong positive correlations with CMT and SCC (r = 0.68–0.91), suggesting that SMT thermography is an effective tool for early detection of SCM [35].

In studies, thresholding, cluster-based algorithms, and more recently semantic segmentation networks such as UNet and DeepLabV3+, as well as YOLO-derived target detection networks (YOLOv3, YOLOv5, EFMYOLOv3, DCYOLO) are used for the automatic detection and segmentation of the breast region in thermal images [26,36,37,38,39,40]

These networks enable the rapid and accurate localisation of areas such as the eye and mammary gland, allowing for the extraction of pixel-based temperature distributions and the classification of mastitis probability; mastitis classification accuracy reaches 83–87%, and sensitivity reaches 80–96% in most studies [26,36,37,39].

In more classical approaches, maximum/mean temperatures obtained from the anterior and posterior mammary quadrants are correlated with somatic cell count (SCC) and California Mastitis Test (CMT) results using regression, ROC analysis, or CART decision trees; it has been shown that temperature–SCC correlation coefficients reach high values such as 0.86–0.97 for the anterior mammary quadrants, and the probability of subclinical mastitis increases significantly above certain threshold temperatures [40,41,42,43].

Some studies report that healthy, subclinical, and clinical mastitis udders can be automatically classified using pixel thresholding and the number of pixels within specific temperature ranges [38,40].

Overall, the literature indicates that there is high potential for estimating and early detection of mastitis rates in cow udders from thermal camera images when appropriate camera settings, standard shooting protocols, and advanced image processing/deep learning models are used; however, environmental factors and a lack of standardisation remain significant areas of research [26,43,44,45].

Although artificial neural networks and thermal imaging have previously been used for mastitis detection, most studies analyse the udder thermal image as a single input without considering spatial temperature variations within the udder. In the present study, a zone-based thermal feature extraction approach was applied, in which each udder thermal image was divided into nine equal regions and thermal characteristics from each zone were analysed separately. This strategy enables the identification of localised temperature anomalies that may occur in specific udder sections during inflammatory processes. By incorporating the relative contributions of different udder zones into the ANN model, the proposed framework aims to reduce the influence of irrelevant regions and improve the detection of mastitis-related thermal patterns under commercial farm conditions.

The primary objective of this study is to detect the risk of mastitis in dairy cattle in real time by utilizing thermal imaging technology. Within the scope of the research, localised temperature increases in the udder region—caused by bacterial infections—will be measured through thermal cameras. These thermal data will then be analysed using Artificial Neural Networks (ANN) to predict the likelihood of mastitis based on somatic cell count (SCC) variations. The proposed early detection system aims to minimize productivity losses and economic damage in dairy farming operations caused by mastitis.

## 2. Materials and Methods

### 2.1. Obtaining Milk Samples and Counting Somatic Cell

Data were collected between March and April 2022 from a commercial dairy farm in the southern Hungary, with a herd of 500 lactating Holstein cows were housed in a free-stall barn, which collaborates with the University of Szeged in academic studies. The enterprise milks its high-yielding cows at 8 h intervals, totalling three sessions per day. The milking unit was maintained within the standard thermal comfort zone for dairy cows, with ambient temperatures ranging between 15 °C and 20 °C and relative humidity levels kept within the 60% to 80% range, as recommended for optimal milking conditions and animal welfare. Data acquisition for this study was conducted during the afternoon milking session. After the cattle were taken to the milking unit, the teat tips were cleaned, and the milking machine/cluster are placed on the udder. To ensure homogeneous milk sampling, milk sampling tubes were attached to the milking unit, and milk samples were collected in these containers during milking. The milk samples collected during milking were taken to the enterprise’s SCC measuring device (Bentley FTS/FCM (Bentley Instruments, Inc., Chaska, MN, USA), an automated flow cytometry-based system capable of rapidly and accurately determining somatic cell counts in raw milk) for analysis.

### 2.2. Acquisition of Thermal Images

The image acquisition process was carefully standardised to ensure consistent and accurate thermal measurements. Following the entry of the cows into the milking unit, a visual inspection of the udders was conducted to assess hygiene status. In cases where visible contamination such as mud or debris was observed, the udder surface was cleaned with a gentle spray of lukewarm water, followed by a two-minute waiting period to allow for proper drainage and moisture reduction. Afterwards, the teat surfaces were wiped with a moist cloth and dried with a clean, dry towel to prevent microbial contamination.

**Figure 1:**
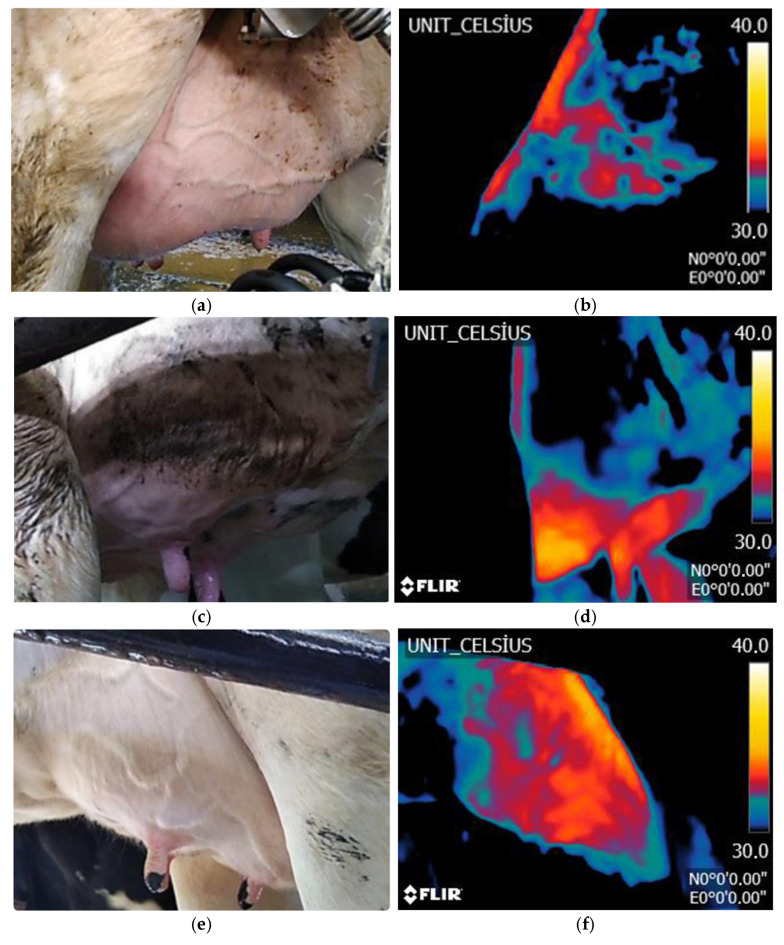

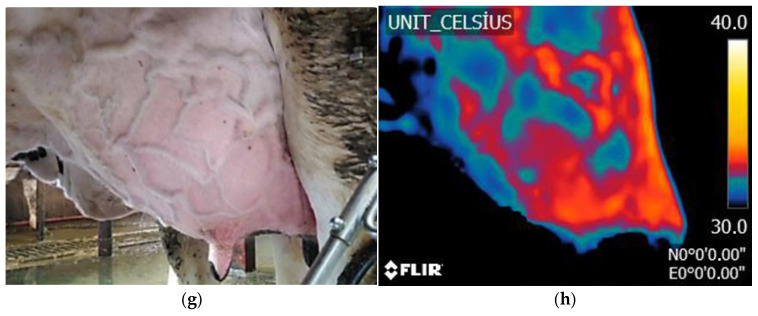
Sample photos by group. (**a**) Negative raw image. (**b**) Negatives thermal image. (**c**) Trace raw image. (**d**) Trace thermal image. (**e**) CMT +1 raw image. (**f**) CMT +1 thermal image. (**g**) CMT +2 raw image. (**h**) CMT +2 thermal image.

After the cleaning procedure, a thermal image of each cow was captured using a FLIR-brand thermal camera (software version 3.9.2) integrated into a Cat S60 smartphone (FLIR Systems, Inc., Wilsonville, OR, USA) (Figure 1). The thermal imaging was performed at an optimal distance of 40–50 cm from the udder to ensure proper image framing. This distance was selected to allow the udder to fit perfectly into the frame using a wide-angle camera, facilitating accurate capture of the entire udder image.

Calibration was performed by taking fixed-point measurements inside the milking unit with both the thermal and infrared cameras, adjusting the thermal camera’s emission settings to match the infrared camera’s results. The system’s accuracy was confirmed with repeated measurements, yielding a deviation of ±0.1 °C. The thermal camera used a resolution of 640 × 480 pixels, providing sufficient detail for the required analysis. The images were captured in a well-lit, indoor environment to ensure consistent image quality and minimize ambient temperature variations.

All images were obtained indoors under controlled lighting and ambient conditions during a single milking session to ensure environmental consistency. Out of an initial dataset of 600 images—comprising one thermal photographs (lateral views) per cow—500 high-quality udder images were selected for further analysis, excluding those with visual noise or distortion. Each image was recorded together with the corresponding cow’s ear tag number to ensure traceability.

In the thermal images presented in Figure 1, the temperature distribution across the udder surface is illustrated using a standardized colour gradient scale ranging from 30 °C to 40 °C. Within this scale, black regions correspond to temperatures below 30 °C, while white regions indicate temperatures approaching 30 °C. This visual representation facilitates the interpretation of spatial temperature variations, enabling the identification of localized thermal anomalies that may be indicative of underlying physiological conditions such as mastitis.

### 2.3. Preparation of Data and Statistical Analysis

**Figure 2:**
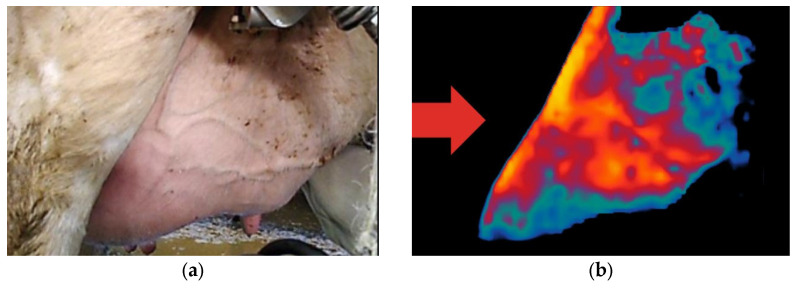
Raw image and processed image. (**a**) Standard camera image; (**b**) Thermal camera image.

Thermal camera images were digitised using image processing methods implemented in the FLIR Thermal Studio Suite 2021 software and used as input data for correlation with somatic cell counts. Each image was cropped to a standardised size of 300 × 300 pixels to ensure consistency in the analysis (Figure 2).

To ensure accurate thermal analysis focused solely on the udder, each 640 × 480-pixel thermal image was manually cropped using Adobe Photoshop to a fixed resolution of 300 × 300 pixels, capturing only the udder region. This dimension was chosen to preserve relevant thermal data while optimising the computational performance of image processing tasks. The resulting photographs are cut in size of 300 × 300 pixels (Figure 2).

In order to avoid anything other than udders in the photograph, areas outside the udder were converted into black pixels and removed using image processing procedures in Adobe Photoshop.

**Figure 3:**
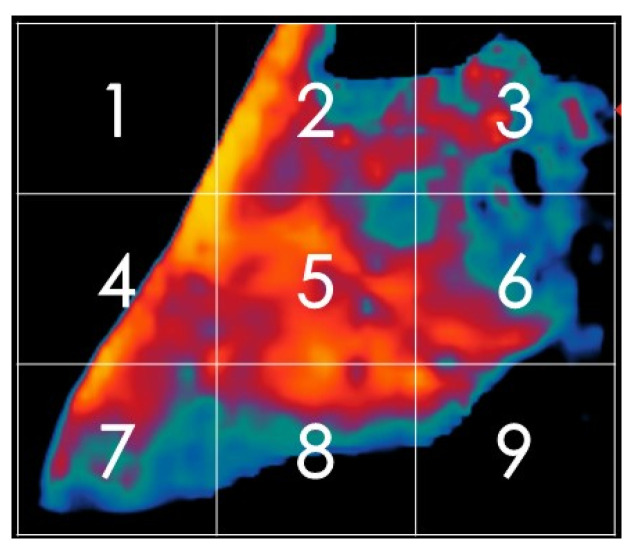
Thermal images are divided into nine zones.

Subsequently, each 300 × 300 image was divided into nine equal zones (Figure 3) to extract localised thermal information. This segmentation approach aimed to minimise the influence of unrelated regions and enhance the model’s ability to detect temperature anomalies within distinct udder sections. The decision to use nine regions was based on a trade-off between spatial resolution and model complexity, ensuring efficient yet informative data extraction.

**Figure 4:**
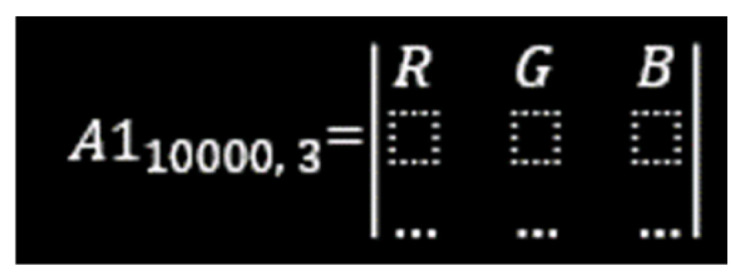
Creating a 10,000 × 3 matrix.

In this process, each thermal image was divided into nine equal segments, with each segment having a resolution of 100 × 100 pixels. All images were originally in RGB (Red, Green, Blue) format, meaning each segment contained three colour channels, resulting in a data structure of 100 × 100 × 3 (i.e., 10,000 × 3) per segment. These pixel values were subsequently reshaped into a two-dimensional matrix of size 10,000 × 3 for further computational analysis (Figure 4).

**Figure 5:**
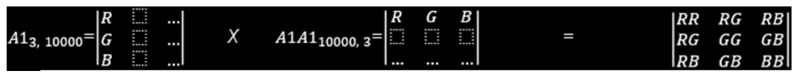
Matrix multiplied by transpose.

The matrix multiplied by the matrix with its transpose (Figure 5).

After the matrix multiplication, the resulting 3 × 3 matrix contained symmetric duplicated values (Figure 5). To avoid redundancy, the upper triangular elements (positions 2, 3, and 6) were removed, and six distinct features were retained from each segment. The same procedure was applied to all nine segments, and the resulting values were concatenated to form a single feature vector of size 1 × 54 for each image. Applying this process to 500 thermal images yielded a final input matrix of dimensions 54 × 500, which served as the dataset for subsequent analysis.

The SCC in the dataset ranged from 0 to 5 million cells/mL. Due to the wide variability of these values, a direct regression approach would be impractical; therefore, the data were categorised into classes based on the CMT scoring system (Table 1).

The target matrix is specified as 1 × 500 in 4 different classes (Table 1). It should be noted that the dataset used in this study exhibits a certain degree of class imbalance. As shown in Table 1, the number of animals in each CMT score group is not equal. Specifically, the dataset contains 217 animals in the negative group, 158 in the Trace group, 83 in the CMT +1 group, and 42 animals in the CMT +2 mastitis group. This distribution reflects the natural occurrence of mastitis cases in dairy herds, where severe infections are relatively less frequent than healthy or mildly affected cases.

To preserve the real-world characteristics of the dataset, no artificial resampling techniques such as oversampling or under sampling were applied. Instead, the performance of the proposed model was evaluated using class-specific metrics and confusion matrix analysis to ensure that the classification performance for minority classes was properly assessed.

**Figure 6:**
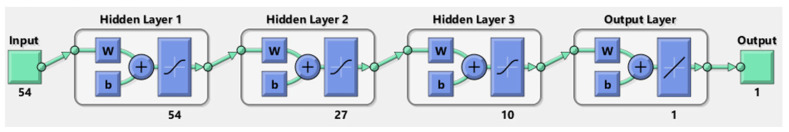
ANN model.

The lateral views of the thermal images were incorporated into the model as 54 input variables, while the SCC was defined as the target output variable (Figure 6).

In addition to the coefficient of determination (R^2^), several classification performance metrics were used to evaluate the diagnostic capability of the artificial neural network model. Since mastitis grading represents a multi-class classification task, commonly used metrics including overall accuracy, precision, recall, and F1-score were calculated for each class. These metrics provide complementary information about the model’s classification performance by quantifying the proportion of correctly predicted samples and the balance between false positives and false negatives.

Furthermore, confusion matrices were generated to visualize the classification results and identify potential misclassifications between mastitis grades. To facilitate interpretation, normalised confusion matrices were also constructed by dividing each row by the total number of samples in the corresponding class.

The artificial neural network (ANN) model used in this study was implemented as a feedforward backpropagation network. Since there is no predefined optimal architecture for artificial neural networks in this context, several network topologies with different numbers of hidden layers and neurons were experimentally evaluated (Table 2) to determine the most suitable structure for the dataset.

The dataset was randomly divided using MATLAB’s (version 2021) default data division procedure into three subsets: 70% for training, 15% for validation, and 15% for testing. The training subset was used to update the network weights, while the validation subset was employed to monitor the generalization performance of the network during training through the early stopping mechanism in order to prevent overfitting. The independent test subset was used exclusively for the final evaluation of the trained model.

The network was trained using the Levenberg–Marquardt backpropagation algorithm (trainlm) with the mean squared error (MSE) as the performance function. Hyperbolic tangent sigmoid (tansig) activation functions were used in the hidden layer(s). In contrast, a linear activation function (purelin) was applied in the output layer to accommodate the regression-based output representation.

Since mastitis grading represents ordered categories, the network produced continuous outputs, which were subsequently mapped to discrete mastitis grades using predefined threshold intervals to perform classification-based evaluation.

The tested network architectures included 10–1, 27–10–1, 54–27–10–1, and 108–54–27–10–1. The performance of each architecture was evaluated using the coefficient of determination (R^2^). The obtained R^2^ values were 0.8533, 0.7991, 0.9166, and 0.2609, respectively (Table 2). The architecture with four hidden layers resulted in a substantial decrease in model performance, likely due to overfitting and increased model complexity relative to the dataset size. Among these configurations, the architecture with three hidden layers consisting of 54, 27, and 10 neurons achieved the highest predictive performance (R^2^ = 0.9166) and was therefore selected as the optimal network structure for the final model. The network was trained using a learning rate of 0.01 over 1000 epochs.

To account for the stochastic nature of artificial neural networks, where varying random initialisations may lead to different optimisation paths and outcomes, the model training and evaluation process was repeated 10 times across independent executions. This multi-run approach was adopted to obtain a more reliable and statistically robust estimate of model performance.

The overall model performance is reported as mean ± standard deviation of the obtained metrics. These metrics are presented separately for the training, validation, and test sets to enable a comprehensive evaluation of model generalisation and potential underfitting or overfitting behaviour.

Additionally, the best-performing model, identified based on validation performance, is reported separately to illustrate the maximum achievable performance under the specified configuration. This model is presented for reference purposes and does not represent the expected general performance.

## 3. Results and Discussion

**Figure 7:**
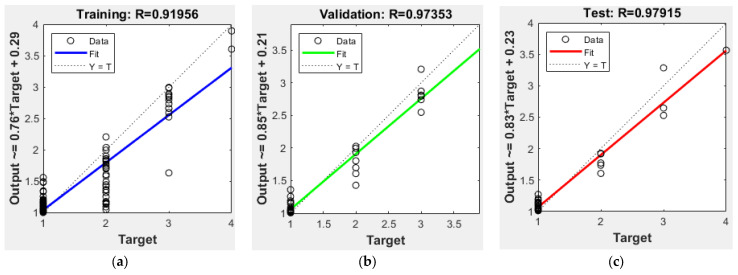
ANN results in regression graphs. (**a**) Training. (**b**) Validation. (**c**) Test.

The data were divided into three subgroups: training (70%), validation (15%), and testing (15%). The correlation coefficient (R) between the estimated and reference target data were 0.91, 0.97, and 0.97 for the training, validation, and test datasets, respectively. The close agreement between the validation and test results indicates robust generalisation capability and the absence of overfitting (Figure 7).

In some runs, test performance exceeded training performance. This behaviour may suggest potential underfitting, where the model may not be sufficiently complex to fully capture the underlying patterns in the training data [46,47]. Alternatively, this discrepancy can also be attributed to variability introduced by data partitioning and the relatively limited sample size, which may lead to fluctuations in performance estimates across different subsets [48,49].

The use of multiple independent runs and reporting results as mean ± standard deviation reduces the risk of misinterpretation arising from such variability and is widely adopted in machine learning experiments [50,51]. Overall, these findings indicate that the model demonstrates reasonably stable generalisation performance, although further optimisation of model complexity and increased dataset size could improve training convergence and reduce variability between training and test results [46,47].

For the training, validation, and test datasets, the ANN model’s average performance values were 0.8862 ± 0.072, 0.9515 ± 0.029, and 0.9481 ± 0.057, respectively (Table 3). The model generalises well without significant overfitting, as indicated by the close agreement between validation and test performance. In some cases, validation and test performance exceeded training performance, suggesting mild underfitting or attributable to variability arising from data partitioning and the relatively limited sample size. The comparatively low standard deviation observed in the validation set further supports the model’s stability across multiple runs. Furthermore, while the best-performing model achieved a test performance of 0.97915, representing the upper bound under the specified configuration, the averaged results provide a more reliable estimate of the expected model performance.

**Figure 8:**
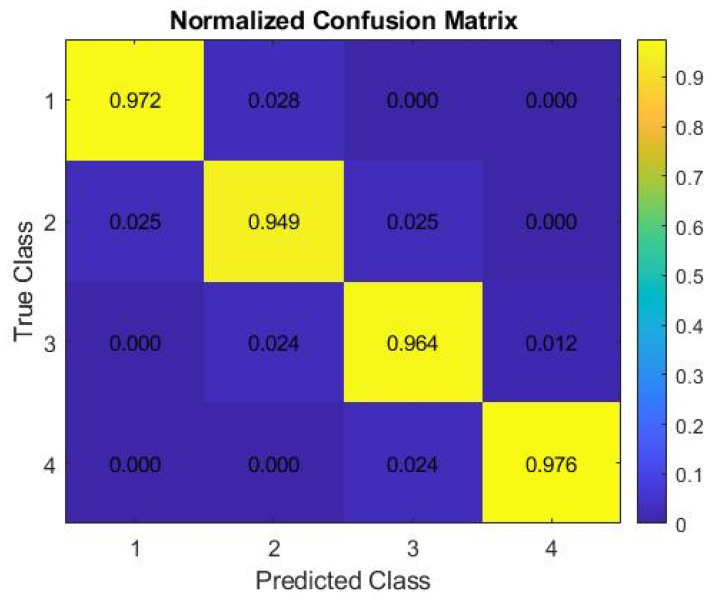
Normalised confusion matrix of model predictions.

**Figure 9:**
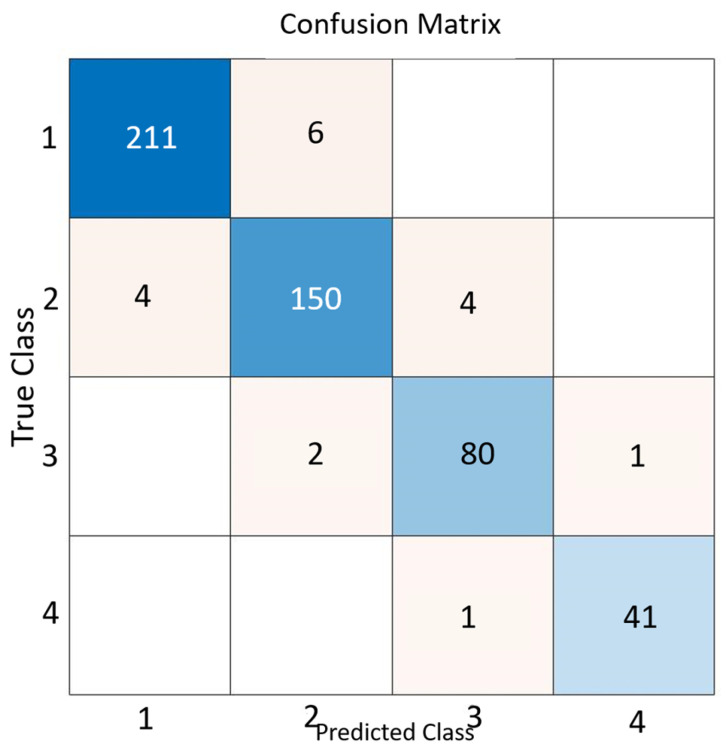
Confusion matrix for model predictions.

The developed ANN model exhibits high performance in classifying CMT scores across four categories (Negative, Trace, +1, and +2) (Table 4). When evaluating the confusion matrix (482/500 correct classifications, 96.4% accuracy) alongside class-based metrics (precision, recall, and F1-score ≥ 0.94 for each class) (Figure 8 and Figure 9), it is evident that the model is robust and well-balanced in terms of both overall and class-specific success criteria. Notably, the precision and recall values of ≈ 0.98 for the +2 (serious) and Negative classes are significant, as they minimise the risk of clinically critical misclassifications.

The CMT-based classification model developed in our study demonstrates high accuracy and balanced inter-class performance in distinguishing between clinical and subclinical forms of mastitis (overall accuracy ≈ 96.4%). This result is consistent with the extensive literature highlighting the critical importance of early and accurate diagnosis of mastitis for herd health and reducing economic losses. It is frequently emphasised that mastitis leads to significant economic losses through decreased milk yield and quality, treatment and labor costs, and increased herd shedding; and that subclinical mastitis, while constituting the majority of cases, can remain a source of infection within the herd without showing clinical signs [52,53]. Therefore, the automatic, rapid, and less error-prone classification of CMT scores is an important tool for both early warning and herd management.

Subclinical mastitis occurs at a considerably higher rate than clinical mastitis, and elevated milk somatic cell counts (SCC) have been shown to negatively impact milk yield, composition, and the metabolic and immune status of dairy cows [52]. Modulation of the rumen microbiota has been demonstrated to alleviate subclinical mastitis, reducing both SCC and proinflammatory cytokine levels [52]. In this context, the CMT-based ANN model’s ability to accurately classify subclinical categories such as Trace and +1 directly supports the literature-reported goal of early intervention during the subclinical stage. Moreover, under real farm conditions, CMT is routinely employed as a daily screening tool, and when combined with real-time monitoring of rumen temperature, it enables early detection of subclinical mastitis [53]. Automated CMT classification can thus be integrated into sensor-based early warning systems to enhance herd health management.

Today, in modern enterprises (where milking is done with computerized herd management systems), characteristics such as milk yield, milk flow rate, and electrical conductivity are automatically recorded during milking. It is reported in the program that cows showing excessive deviation in electrical conductivity may have mastitis [54,55,56]. However, it is often found that these alarms are false and that diagnosing mastitis using only electrical conductivity [55,57,58,59,60] is not reliable [61].

Artificial neural networks have been widely used for mastitis detection due to their ability to model complex nonlinear relationships among biological variables. Singh et al. [62] reported a very high classification accuracy (0.99) using ANN models based on physical udder characteristics for the detection of clinical mastitis. Similarly, Lopez-Benavides et al. [63] applied artificial neural networks using electrical conductivity, somatic cell count (SCC), fat percentage, and protein percentage as input variables, achieving an accuracy of 0.83 with a multilayer perceptron model. In the present study, the ANN model achieved an overall classification accuracy of 96.4%, with precision, recall, and F1-score values exceeding 0.94 across all CMT score categories. Notably, very high performance was observed for the negative and serious (+2) classes (F1-scores of 0.976), while slightly lower but still strong performance was obtained for the subclinical categories.

The high classification performance observed in this study may be attributed to the ANN model’s ability, trained with the Levenberg–Marquardt backpropagation algorithm, to effectively capture nonlinear relationships among the input variables. In addition, the use of hyperbolic tangent-sigmoid activation functions in the hidden layer(s) likely enhanced the network’s ability to learn complex patterns in the dataset. Compared with previous studies, the performance in the present study is particularly noteworthy because the model was designed to perform multi-class classification of mastitis severity, including subclinical conditions, rather than distinguishing only clinical mastitis cases. Since subclinical mastitis accounts for the majority of mastitis cases and is more difficult to detect under field conditions, the ability of the proposed ANN model to successfully differentiate multiple CMT score categories underscores its potential for early detection and monitoring of mastitis in dairy herds.

Several previous studies have evaluated the performance of artificial neural networks for mastitis detection using different types of biological and sensor-derived data. For example, Heald et al. [30] developed an ANN model based on individual test-day milk parameters and compared its performance with linear discriminant analysis. Although ANN outperformed the linear discriminant approach, the maximum classification accuracy reported in that study was 0.71. Similarly, Grodkowski et al. [64] investigated early mastitis detection using a monitoring system based on 3D motion sensors and reported comparable predictive performance for both ANN and logistic regression models, with accuracies of approximately 0.70. In contrast, Cavero et al. [65] used variables such as electrical conductivity, milk yield, milk flow rate, and days in milk as ANN inputs; however, the resulting model demonstrated limited predictive capability, achieving an accuracy of only 0.49.

Compared with these studies, the ANN model developed in the present work achieved a substantially higher overall classification accuracy (96.4%), together with consistently high precision, recall, and F1-score values across the CMT score categories. The model showed particularly strong performance in identifying both healthy samples and severe mastitis cases, while also maintaining robust discrimination among subclinical classes. These findings indicate that the modelling framework applied in this study was effective in capturing diagnostically relevant patterns within the dataset.

Differences in predictive performance among studies may arise from several factors, including variations in the structure and quality of the datasets, the types of input variables used, and methodological developments in machine learning techniques over time. Consequently, the results obtained in the present study suggest that the proposed ANN framework provides a reliable and effective approach for mastitis classification under the evaluated conditions.

## 4. Conclusions

The results of this study indicate that artificial neural networks (ANNs) applied to thermal camera images can effectively detect mastitis. Preliminary findings demonstrated that dividing the image into nine udder regions improved the ANN’s ability to localise temperature-related inflammation. Manual segmentation allowed the model to focus on diagnostically significant zones, thereby improving classification accuracy. Moreover, a 30–40 °C colour mapping also outperformed other temperature ranges, offering higher visual contrast for identifying mastitis-related thermal variations.

To increase prediction accuracy, additional variables can be incorporated into the input data, such as the number of lactations, milk yield, and the age of the animal, as well as daily individual-animal measurements. However, environmental changes can influence the results. By comparing output values obtained from measurements on different days, the impact of environmental variation can be minimised, thereby improving the accuracy of predictions.

However, this study has certain limitations. The model was trained using a limited number of thermal images under relatively controlled environmental conditions, which may restrict its generalisability. Moreover, factors such as feed composition, udder conformation, and ambient humidity were not considered and could influence the outcome.

In future studies, expanding the dataset with longitudinal records and integrating real-time sensors (e.g., humidity, air velocity) may significantly enhance the robustness of the system. Furthermore, integrating this detection model into automated herd management software may enable early detection of mastitis in commercial dairy farms, contributing to improved animal welfare and economic efficiency.

## Figures and Tables

**Table 1:** CMT scores SCC table [34] and animal counts.

CMT Score	Mean SCC/mL	Animal Count
Negatives	<200,000	217
Trace (subclinical)	200,000–400,000	158
+1 (subclinical)	400,000–1,200,000	83
+2 (serious)	1,200,000–5,000,000	42

**Table 2:** Performance comparison of tested ANN architectures.

Hidden Layers	Network Architecture	Epoch	R^2^
1	10–1	6	0.8533
2	27–10–1	6	0.7991
3	54–27–10–1	13	0.9166
4	108–54–27–10–1	12	0.2609

**Table 3:** Performance of the ANN model over 10 independent runs. The correlation coefficients (R) between the estimated and references target.

Dataset	Train	Validation	Test
Test Model (mean ± std.)	0.8862 ± 0.072	0.9515 ± 0.029	0.9481 ± 0.057
Best Test Model	0.91956	0.97353	0.97915

**Table 4:** Classification performance of the ANN model.

CMT Score	Precision	Recall	F1-Score
Negatives	0.981395	0.97235	0.976852
Trace (subclinical)	0.949367	0.949367	0.949367
+1 (subclinical)	0.941176	0.963855	0.952381
+2 (serious)	0.97619	0.97619	0.97619

Accuracy: 96.4.

## Data Availability

The data presented in this study are available on request from the corresponding author due to ethical and privacy restrictions related to farm-level data.
